# Pitfalls of predicting age‐related traits by polygenic risk scores

**DOI:** 10.1111/ahg.12520

**Published:** 2023-07-07

**Authors:** Valentina Escott‐Price, Karl Michael Schmidt

**Affiliations:** ^1^ Centre for Neuropsychiatric Genetics and Genomics School of Medicine, Cardiff University Cardiff UK; ^2^ UK Dementia Research Institute Cardiff University Cardiff UK; ^3^ School of Mathematics Cardiff University Cardiff UK

## Abstract

Polygenic risk scores (PRS) are a method increasingly used to capture the combined effect of genome‐wide significant variants and those which individually do not show genome‐wide significant association but are likely to contribute to the risk of developing diseases. However, their practical use incurs complications and inconsistencies that so far limit their clinical applicability. The aims of the present review are to discuss the PRS for age‐related diseases and to highlight pitfalls and limitations of PRS prediction accuracy due to ageing and mortality effects. We argue that the PRS is widely used but the individual's PRS values differ substantially depending on the number of genetic variants included, the discovery GWAS and the method employed to generate them. Moreover, for neurodegenerative disorders, although an individual's genetics do not change with age, the actual score depends on the age of the sample used in the discovery GWAS and is likely to reflect the individual's disease risk at this particular age. Improvement of PRS prediction accuracy for neurodegenerative disorders will come from two sides, both the precision of clinical diagnoses, and a careful attention to the age distribution in the underlying samples and validation of the prediction in longitudinal studies.

## 1 BACKGROUND

Dementia is primarily a disease of ageing, with prevalence much higher in older age groups; among people above the age of 65, dementia prevalence is 1 in 14, while above the age of 80, the prevalence rises to 1 in 6. Moreover, in the United Kingdom, the growth of prevalence of dementia is fastest in the age group above 65 in the United Kingdom according to the Office of National Statistics (ONS, 2021). The most common form of dementia is Alzheimer's disease (AD), accounting for more than 60% of cases, and this is also the most studied form of dementia. Very early AD cases (aged 30–50) are mostly attributed to rare highly penetrant mutations in the *APP*, *PSEN1* and *PSEN2* genes (Chavez‐Gutierrez et al., 2012). The mean age at clinical onset of AD is about 68 in *APOE*‐e4e4 carriers and about 84 in *APOE*‐e4 noncarriers (Liu et al., 2013). Furthermore, Lo et al. (2019) recently investigated age‐related genetic heterogeneity of AD and found only a moderate genetic correlation (*r_g_* = 0.64) between the two age groups considered (60–79 years vs. 80+ years). This indicates a potential difference in the genetic architecture of AD depending on the age at (clinical) onset. In addition to disease (or gene) related mortality, this makes studies of the genetic background of neurodegenerative disorders more complicated than that of neurodevelopmental disorders. Conducting large‐scale genome‐wide association studies (GWAS) in adults employing readily available population‐based controls, with the aim of increasing the statistical power of the study, is not very problematic for neurodevelopmental disorders with small prevalence and an early age at onset such as autistic spectrum disorder (onset in early childhood; see e.g., Tan et al., 2021) or schizophrenia (mean onset ∼30 years of age; see e.g., Gogtay et al., 2011), but in neurodegenerative disorders the age of case and control groups turns out to be of crucial importance.

The success of genome‐wide association studies has led to growing interest in making predictions of complex trait phenotypes and diseases from genotype data. Going beyond the genetic association of the disorder with rare mutations or single highly associated loci, which only explains part of the heritability, the Polygenic Risk Score (PRS) method, where the effect sizes of a moderate or large number of risk and protective alleles are combined into a predictor variable, has shown a great potential to stratify individuals into risk categories based on their genetic profile for common genetic disorders (Escott‐Price et al., 2015; Purcell et al., 2009). While the polygenic risk scores predict an individual's general liability to develop the disease in question within their lifetime, this approach can be taken further to predict particular aspects of the disease by selecting variants occurring in putative disease‐specific pathways (Ahmad et al., 2018; Grama et al., 2020). Thus, PRS in principle offer a route both to understanding the pathways involved in disease pathogenesis and to identifying people in the general population who are at high risk of developing the disease. However, the utility of risk scores in precision medicine and clinical settings remains an open question (Lewis & Vassos, 2017).

In particular, application of PRS for late onset Alzheimer's disease (AD) prediction showed that PRS is a statistically significant predictor of AD risk and of conversion to AD (Bellenguez et al., 2022; Escott‐Price et al., 2015; Fulton‐Howard et al., 2021); however, the PRS was calculated differently in different studies, both in terms of the numbers of variants and of the analytical approaches used. The prediction accuracy measured by area under the curve (AUC) lies in a range of between 70% and 84% (Altmann et al., 2020; Escott‐Price et al., 2017), depending on the study. Rigorous assessment of the value of predictors and the methodology used for PRS generation is critical before implementation. Here we discuss some of the limitations and pitfalls of prediction analysis for age‐related traits and show how naïve implementations can lead to severe bias and misinterpretation of results.

### 1.1 Age effects on PRS

The choices made in conducting a discovery GWAS study are often motivated by expediency and data availability rather than a specific study design. The genetic risk score, which includes only the most highly associated, genome‐wide significant variants from the discovery GWAS (association *p* values less than 5 × 10^−8^), is composed of a fairly small number of variants (e.g., 83 variants; Bellenguez et al., 2022) and therefore potentially misses information from moderately associated variants. In contrast, the use of more relaxed *p*‐value thresholds leads to inclusion of thousands of variants (Leonenko et al., 2021) and will inevitably introduce some random noise. The former approach will mostly reflect the effect of the *APOE* gene, as it has the strongest association with the disease (*β* ≈ 1.2 for rs429358; Kunkle et al., 2019) while the addition of a modest number of variants with effect sizes close to zero (*β* < 0.2; Andrews et al., 2020) will only mildly alter an individual's risk score. Therefore, this risk score will firstly prioritize the risk of *APOE*‐e4 carriers and tend to overlook the risk of *APOE*‐e4 noncarriers. In addition, since e4 carriers also have earlier age at onset, this risk score will give best prediction in a sample of younger participants. For example, in Bellou et al. (2020), the sample was collected at mean age of 64 years and the optimal choice of selection threshold was *p*‐value < 10^−5^. In contrast, in samples of older and/or pathology confirmed individuals, with mean age ∼80 years (Escott‐Price et al., 2017; Leonenko et al., 2021), the best accuracy was achieved when including thousands of variants with a threshold of AD association *p*‐value < 0.1. This age effect can also be observed in the following illustrative example (see table 1 in Bellou et al., 2020). In a sample of age below 80 years, the APOE‐variable (constructed as a sum of numbers of e4 and e2 alleles weighted by their effect sizes) by itself has *β* ≈ 1.15 and achieves prediction accuracy AUC ≈ 0.74, while a PRS constructed from variants outside the *APOE* region has *β* ≈ 0.68 and AUC ≈ 0.68. For age 80 years and above, the relative effect sizes are reversed (*APOE*: *β* ≈ 0.78, AUC ≈ 0.67; PRS: *β* ≈ 0.68, AUC ≈ 0.81). A possible explanation for this is that in the higher age group the *APOE*‐e4 risk allele is already depleted due to AD and other conditions affecting mortality associated with the allele; indeed, the allele frequency of *APOE*‐e4 has been reported to decrease from 0.18 at age 60 to 0.09 at age 90 in individuals of European ancestry (McKay et al., 2011). Although the *APOE* effect size decreases in older cohorts, the highest AD prevalence is reported in individuals over 80 years of age (Hebert et al., 2003); this indicates that other genes with smaller effect sizes contribute essentially to the disease risk. Therefore, polygenic factors are likely to play a higher role in AD at higher age. This age‐dependent shift in the relative importance of single‐gene and polygenic factors complicate the application of PRS. Using *APOE* and a PRS calculated excluding the *APOE* region as two separate predictors in bivariate regression here gives a consistent result with prediction accuracy exceeding both separate predictors in both age ranges (AUC ≈ 0.80 for age below 80 years, AUC ≈ 0.82 for 80 years and above; Bellou et al., 2020).

**FIGURE 1:**
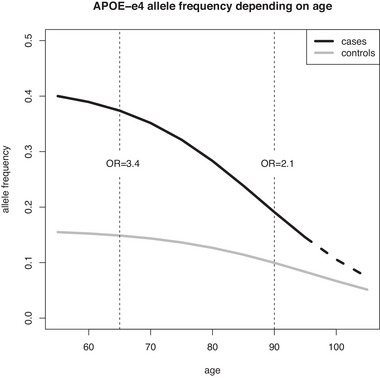
APOE‐e4 allele frequency dynamics in an ageing population.

In age matched samples of cases and controls, the strong genetic risk factors, such as *APOE*‐e4 for AD, are likely to remain statistically significant and maintain the same direction of association but show decreasing effect size as the age in the samples increases. Figure 1 schematically shows the *APOE*‐e4 allele frequency dynamics in an ageing population, with frequency *f* = 0.42 in AD cases (age at onset 66.4 ± 7.8 years) and *f* = 0.16 in population controls (age 66.0 ± 6.5 years), with OR consistently reported as ≈ 3.4 (see e.g., the review by Andrews et al., 2020). The *APOE*‐e4 frequency is nearly halved (*f* = 0.086) in centenarian controls (age 101.4 ± 1.3 years) (Tesi et al., 2019). Farrer et al. (1997) report an apparent risk of the *APOE*‐e4 allele with OR ≈ 2 at age 90 which could not be reliably assessed after age 95 (see Figure 1). In addition, the heritability explained by variants on chromosome 19 (which harbors APOE) was significantly larger in the younger age group (below 80 years of age at onset) than in the older (above 80 years of age at onset) (Lo et al., 2019). The authors also demonstrated that other genes with genome‐wide significant association with AD (*BIN1* and *PICALM*) show larger effects at younger age (Lo et al., 2019).

Like the effect illustrated in Figure 1, in a plausible scenario for a variant with an association effect size OR ≈ 1.15, as typical for a GWAS, its effect size OR will also decrease to or even below 1. In particular, if the disease risk allele frequency in the ageing population decreases faster in cases than in controls (for similar reasons as in the case of *APOE*), then even in a comparison of cases and controls within the same age group, the direction of association may eventually be reversed.

Finally, when older cases, that is, individuals that reached a sufficiently high age to develop a neurodegenerative condition, are compared with *younger controls*, genes which are related to other diseases with earlier onset and, consequently, earlier mortality (e.g., diabetes, cancers, stroke, etc.) may spuriously appear to be associated with the neurodegenerative disorder.

### 1.2 Genetic background effects on PRS

The determination of variant effect sizes in GWAS faces the problem that the diagnostic accuracy for Alzheimer's disease and other dementias is generally poor (Beach et al., 2012; Escott‐Price et al., 2019), introducing uncertainty into the observed odds ratios. Moreover, it is now common practice to perform AD GWAS, more appropriately considered as AD‐related dementia GWAS, using dementia proxies based on family history in the UK Biobank as putative cases and population‐based young unscreened controls. This allows for larger GWAS that would be expected to have higher statistical power, but in fact provide less reliable estimates of effect sizes compared to smaller, more precisely screened GWAS, resulting in a smaller fraction of explained heritability (Escott‐Price & Hardy, 2022). As the PRS is determined from a moderate to large number of *β* coefficients, their random errors add up to a large uncertainty in the PRS, detracting from its usability.

In addition, allele frequencies and LD patterns vary between different human populations, so an individual's risk score must be calculated and put in relation with a suitable genetic background population. We remark that this also refers to subpopulations such as age groups with different allele frequencies (see discussion of age dependency above). To estimate the disease risk of a particular individual from a particular population, their PRS needs to be considered in the context of a matching background sample. Usually, the obtained PRS distribution is standardized (i.e., rescaled so that scores have mean = 0 and standard deviation = 1) within that sample; however, the standardization parameters will differ in different samples. This introduces some uncertainty in the classification of the individual's risk score as either unremarkable or indicative of a heightened risk of disease. In non‐European samples, the lack of AD genetic research performed in populations of non‐European ancestry further limits the scope of risk prediction by PRS.

### 1.3 Effects of PRS calculation methodology

While the general idea of constructing a PRS is quite straightforward and perspicuous, the practical calculation of PRS faces a number of challenges that may lead to inconsistent and even contradictory outcomes, in particular when applied to assessing an individual's risk of developing the disease.

The original and simplest way of PRS calculation involves selection of the variants most strongly associated with the disease and subsequent removal of some of these variants when in linkage disequilibrium (LD) with others. The individual log odds ratios (*β* coefficients) for these variants discovered in a GWAS are then added together, weighted with the number of risk alleles in a genotype, to form a risk score for this genotype. The interpretation of the resulting numbers is based on comparison with their standardized distribution in the controls or population. One of the main parameters in this approach is the inclusion threshold for associated variants, raising the question of whether it is more advantageous to include a large number or to focus on more highly associated variants.

This standard method of PRS calculation may miss out on information contained in loci associated with the disease that happen to fall into a region of higher LD. Therefore, more sophisticated statistical methods of accounting for LD in the formation of a PRS have been devised, for example, using Bayesian models (LDpred (Vilhjalmsson et al., 2015), PRS‐CS (Ge et al., 2019), LDAK (Speed & Balding, 2019), and SBayesR (Lloyd‐Jones et al., 2019)), some of which considerably reduce the transparency of the relationship between the raw effect sizes obtained from a GWAS and the resulting PRS (Escott‐Price & Schmidt, 2021). The prediction accuracy of the PRS, measured in terms of the AUC, is quite similar between the various methods in a variety of variant selection scenarios (e.g., AUC between 0.70 and 0.73 for schizophrenia (Ni et al., 2021), and between 0.73 and 0.74 for AD with two predictors, *APOE* and PRS without *APOE* region, the latter calculated with different methods; see supplemental table 2 in Leonenko et al., 2021). So taken at face value any one of these methods would appear as good as the other; however, it turns out that the choice of method has a crucial effect on the assessment of an individual's PRS score against the population distribution. Indeed, individuals with an extreme PRS value as calculated by one method may have an unremarkable PRS value near the middle of the distribution when another method is used (Leonenko et al., 2021; supplemental figure 5). This ambiguity is a major obstacle to clinical use of PRS until a reliable and correct method of calculating the PRS is identified. As the sample prediction accuracy of the methods is comparable, this identification cannot be based on AUC alone, but will need to take into account the deduction logic of the method and require the assessment of individual prediction correctness in longitudinal studies.

## 2 CONCLUSIONS

Age plays a critical role in the analysis of the genetic background of AD and gives rise to serious complications in the application of the PRS method and the interpretation of the results. There is a strong indication that the genetic architecture of AD is different depending on the age at (clinical) onset. Also, the depletion of risk alleles through mortality, either by AD or by other diseases with which the alleles are associated, leads to substantial shifts in allele frequencies and effect sizes that make PRS scores not comparable between different age groups and may in extreme cases imply apparent reversal of the direction of association and lead to the identification of spurious protective alleles. Imperfections in the underlying GWAS, such as misclassification of cases, use of putative case proxies and lack of attention to the change in genetic characteristics (specifically allele frequencies) in the population with age enter the calculation of the PRS and impact the achievable prediction accuracy. These difficulties are compounded with the more general issues of inconsistency between different available methods of PRS calculation and lack of suitable population studies. Even a PRS in which these issues have been resolved will need to be based on a GWAS focused on a particular age group and subphenotype, and then will give a risk prediction for individuals of lower age to develop that particular subphenotype when they enter this age group.

### 2.1 Future directions for PRS: nonrisk GWAS and their implications for prediction

In view of their fundamental function in the calculation and use of PRS, it will be essential to improve on GWAS study designs in a way that takes into account the inhomogeneities mentioned above. The aim will be to understand not only the clinical heterogeneity of AD, but also the underlying genetic architecture, and to relate specific biological mechanisms (e.g., described by genetic pathways) to specific subphenotypes. Some subphenotypes may be predicted with aid of disease risk GWAS, whereas others may require phenotype specific GWAS. For example, for Huntington's disease, which is caused by a CAG repeat expansion in the Huntingtin gene, *HTT*, genome‐wide association studies of Huntington's disease progression (Moss et al., 2017) and age at onset of motor signs (Correia et al., 2015) have reported novel genetic variants associated with the disease subphenotypes rather than overall risk. Similarly, it has been observed that the rate of cognitive decline in AD is often reported as not associated to *APOE* (Katzourou et al., 2021), however no powerful GWAS of rate of decline in AD exist as yet to generate a PRS for rate of decline prediction. PRS derived from GWAS using biomarkers of neurodegeneration such as amyloid positron emission tomography (amyloid‐beta PET) (Yan et al., 2021) or plasma biomarkers (Bradley et al., 2023; Stevenson‐Hoare et al., 2022) may have more useful applications for treatment, especially as newer therapies are developed which have very specific mechanisms of action (e.g., anti‐amyloid antibodies).

Utilization of subphenotype‐specific pathways is also limited as the pathways are not well defined and their definitions usually rely on literature biased toward positive results and hypothesis driven analyses (Sierksma et al., 2020). Therefore, novel experiments are required to underpin the refined definition or even redefinition of gene‐networks and pathways.

Finally, the disease diagnosis needs to be improved and agreed upon. Today biomarkers may aid in the diagnosis of subtypes of the disease, some more accurately than others (Stevenson‐Hoare et al., 2022). A combination of genetics, biomarkers, brain imaging and other clinical information will provide more accurate analyses, eventually moving away from generic dementia prediction toward a prediction of a specific type of dementia such as Alzheimer's disease, frontotemporal dementia, dementia with Lewy bodies, vascular dementia, etc., and the related phenotypes, for example, age at onset, disease progression, and rate of decline.

## AUTHOR CONTRIBUTIONS

Both authors equally contributed to the design of the study and interpretation of the data, drafted and reviewed the manuscript and approved the final version.

## CONFLICT OF INTEREST STATEMENT

The authors declare no conflict of interest.

## Data Availability

Data sharing not applicable to this article as no datasets were generated or analyzed during the current study.
